# Mouse lung mechanical properties under varying inflation volumes and cycling frequencies

**DOI:** 10.1038/s41598-022-10417-3

**Published:** 2022-05-02

**Authors:** K. A. M. Quiros, T. M. Nelson, S. Sattari, C. A. Mariano, A. Ulu, E. C. Dominguez, T. M. Nordgren, M. Eskandari

**Affiliations:** 1 Department of Mechanical Engineering, University of California, Riverside, CA USA; 2 BREATHE Center, School of Medicine, University of California, Riverside, CA USA; 3 Division of Biomedical Sciences, School of Medicine, University of California Riverside, Riverside, CA USA; 4 Department of Environmental and Radiological Health Sciences, Colorado State University, Fort Collins, CO USA; 5 Department of Bioengineering, University of California, Riverside, CA USA

**Keywords:** Biomedical engineering, Biophysics

## Abstract

Respiratory pathologies alter the structure of the lung and impact its mechanics. Mice are widely used in the study of lung pathologies, but there is a lack of fundamental mechanical measurements assessing the interdependent effect of varying inflation volumes and cycling frequency. In this study, the mechanical properties of five male C57BL/6J mice (29–33 weeks of age) lungs were evaluated ex vivo using our custom-designed electromechanical, continuous measure ventilation apparatus. We comprehensively quantify and analyze the effect of loading volumes (0.3, 0.5, 0.7, 0.9 ml) and breathing rates (5, 10, 20 breaths per minute) on pulmonary inflation and deflation mechanical properties. We report means of static compliance between 5.4–16.1 µl/cmH_2_O, deflation compliance of 5.3–22.2 µl/cmH_2_O, percent relaxation of 21.7–39.1%, hysteresis of 1.11–7.6 ml•cmH_2_O, and energy loss of 39–58% for the range of four volumes and three rates tested, along with additional measures. We conclude that inflation volume was found to significantly affect hysteresis, static compliance, starting compliance, top compliance, deflation compliance, and percent relaxation, and cycling rate was found to affect only hysteresis, energy loss, percent relaxation, static compliance and deflation compliance.

## Introduction

Globally, 600 million people live with chronic lung disease (e.g., asthma, chronic obstructive pulmonary disease (COPD), bronchiectasis) which can increase the risk of other health problems and results in 4 million premature deaths a year and rising^1–5^. This does not include recent deaths associated with the global COVID-19 pandemic which, as of September 2021, surpasses 4.6 million deaths^6^. Diagnosis and treatment of these pulmonary diseases is a massive economic burden; for example, only COPD in the United States alone, is predicted to cost $800 billion in direct medical bills over the next 20 years^7,8^. It is known that pulmonary diseases can change the structure of the lung and in turn alter its mechanical properties^9–12,56^; for example, obstructive diseases, such as asthma and chronic bronchitis, interrupt the airflow due to smooth muscle thickening, mucosal growth, and loss of lung elastic recoil^13–15^. Investigating fundamental pulmonary mechanics can offer avenues to improve our understanding of lung physiology, inaugurate new diagnostics, and assess treatment to alleviate morbidity and mortality, as well as the financial burden imposed by pervasive pulmonary diseases^16,17^.

Mice are widely used in pulmonary research given the readily controllable experimental environment and similarity between murine and human immune systems and physiology^19^. Despite structural differences between species, murine studies include the examination of agricultural dust exposure, epithelial injury, and nicotine to better understand the possible effects on humans^18,20–22^. While investigations of mouse lung mechanics are challenging due to the small specimen size, developments in accurate measuring techniques have improved pressure–volume mechanical characterizations^18,23–26^. Given the importance and translational potential of murine lung research, coupled with capabilities offered by new measurement systems, a comprehensive examination of mechanical energy and elasticity properties will help to advance the state of pulmonary science.

To address this need, we build upon prior work in our group by utilizing our previously validated custom-designed electromechanical apparatus, which continuously delivers controlled air volumes and measures the corresponding pressures and change in volume of the lung specimen^26^. As such, this study offers an examination of whole murine lung viscoelasticity, enabling valuable insights given the energetic modifications demonstrated in diseased states that enable model building and identification^27,28^. To add to the understanding of viscoelastic trends in healthy specimen, comprehensively evaluating pulmonary mechanical properties under interdependent loading amplitudes and cyclic frequencies facilitates baseline measurements and sets the foundation for investigating the root cause of pathologies. Previous investigation of murine mechanics in our group has briefly explored the interconnectivity of maximum inflation volume and inflation rate on a non-statistically significant sample size (n = 2) prompting further study^26^. To the best of our knowledge, no study has examined the simultaneous effects of inflation volume and clinical cycling rate on mouse lung mechanics until now.

Furthermore, the mouse, among other mammalian species, develops a double sigmoidal pressure–volume shape when the inflation pressure is high with the transition occurring past 20 cmH_2_O^29^. Inflating past this pressure transition is common in murine studies focusing on the mechanics of the “full-range” pressure-volume (PV) curves collected from degassed lungs inflated to maximum capacity (~ 35cmH_2_O)^24,25^. This present experimental study also seeks to investigate lung mechanics beyond 20 cmH_2_O inflation and further explore the potentially differing mechanics of inflating above and below 20 cmH_2_O by comparing PV curves across a range of volumes (0.3–0.9 ml). The range used in this study reaches the proposed max inflation or total lung capacity of the mouse (~ 0.85–0.95 ml), a range which has been historically difficult to accurately determine^30^. Covering the full lung volume range facilitates our understanding of how this shape alteration impacts pulmonary mechanics and the recruitment and decruitment of lung units.

This study comprehensively analyzes murine mechanics as characterized by alveolar recruitment and decruitment compliances, hysteresis, and stress relaxation under varying inflation volumes and breathing rates. Results are compared with existing trends from disparate observations in previous studies and across various animals.

## Methods

Five male C57BL/6J mice (31.3 ± 4.5 g) 8–12 weeks of age^31^ were obtained from the Jackson Laboratory (Bar Harbor, ME, USA) and housed in micro-isolator cages at the University of California Riverside animal vivarium. All such experiments and procedures were approved by the UC Riverside Institutional Animal Care and Use Committee (protocol #20200014) and executed in accordance with institutional guidelines and regulations. Mice were allowed unlimited access to food and water, weighed weekly, and monitored for any behavioral or physiological changes. Mice were part of a larger exposure study and served as the healthy control group; as such, they received 1X phosphate buffered saline (PBS) intranasally thrice weekly for 21 weeks. Mice were sacrificed by anesthesia overdose via inhalation: exposure to 5 ml of isoflurane on a cotton ball was initially used followed by cervical dislocation and bilateral thoracotomy to ensure death. Then a 20-gauge cannula was inserted into the trachea and secured with thread. A 1 ml syringe was used to inflate the lung with 0.5 ml of air to prevent the airway collapse during dissection. After the lung was removed from the chest cavity, it was stored in 1XPBS until testing commenced 3 h postmortem. The study is reported in accordance with ARRIVE guidelines.

**Figure 1:**
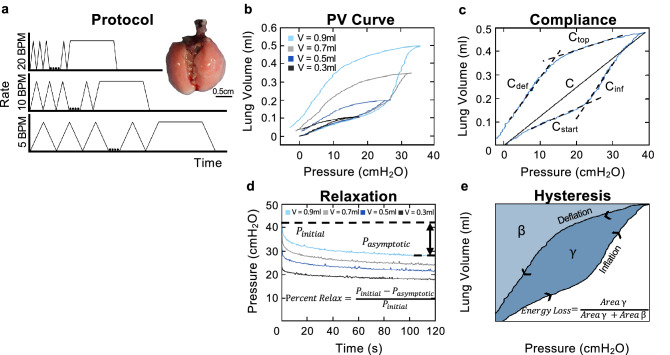
(**a**) Experimental protocol of applied volume at three cycling rates (5, 10, and 20 BPM). Three cycles of preconditioning are implemented followed by an analyzed inflation-deflation cycle and inflation cycle with hold for assessing the viscoelastic response. (**b**) Representative specimen’s quasi-static PV curve at 0.3, 0.5, 0.7, and 0.9 ml (black, dark blue, grey, light blue respectively) illustrates variations in PV curve shape with increasing applied volume. (**c**) Quasi-static PV curve (blue solid line) demonstrating four calculated slopes via linear regression (C_start_, C_inf_, C_top_, C_def_, dashed black) and static compliance (C, solid black). (**d**) Viscoelastic response demonstrating percent relaxation calculation at applied volumes of 0.3, 0.5, 0.7, and 0.9 ml (black, dark blue, grey, light blue respectively). (**e**) Qualitative quasi-static PV curve demonstrating the calculation of hysteresis as area γ and energy loss as the normalized ratio between area γ and the sum of area γ & β.

Each lung was placed inside the tank of our validated custom-designed apparatus to generate pressure–volume (PV) curves by recording continuous pressure and compressed lung volume while controlling applied air volume. For details regarding the system, the authors direct the reader to previous work establishing the apparatus and methodology^26^. Utilizing a similar testing protocol as our previous study, for each test, a preload pressure of 5 cmH_2_O was applied to achieve a shared datum state across all samples, followed by three preconditioning cycles of inflation/deflation to specified inflation volumes, a reset of piston position, a test cycle of inflation/deflation to the same specified inflation volume, and a 120s viscoelastic hold (Fig. 1A)^26,32^. Four volumes (0.3, 0.5, 0.7, 0.9 ml) at three breathing rates (5, 10, 20 breaths per minute, BPM) were considered, totaling twelve positive pressure ventilation tests (Fig. 1B). To minimize air trapping, the testing was sequential, increasing volume and decreasing frequency throughout the test day^33^, such that the three frequencies were tested at 0.3 ml in decreasing order, followed similarly by 0.5, 0.7, and 0.9 ml. To allow tissue reset, testing sequences were separated by two minutes^34^.

Static compliance (C), or chord compliance, was calculated as the ratio of volume to peak transpulmonary pressure, as previously defined^35^. As shown in Fig. 1C and consistent with the labeling of Takeuchi et al.^36^, starting compliance (C_start_) was the compliance measured as the slope of the start to inflation; inflation compliance (C_inf_) was measured as the slope of the most compliant region of the curve and was calculated for inflation volumes of 0.7 and 0.9 ml due to the absence of this region at lower volumes; top compliance (C_top_) was measured as the slope at the beginning of the deflation limb before the point of maximum curvature; and deflation compliance (C_def_) was measured as the slope at the end of the deflation limb of the PV curve. The slopes were calculated via previously outlined methods of linear regression by individually adding points until R^2^ was between 0.97–0.99 to fit the inflation and deflation regions^37^ (MATLAB, MathWorks Inc., Natick, MA, USA).

Peak pressure was found as the maximum transpulmonary pressure at the end of inflation. Relaxation during the viscoelastic hold was calculated as the percentage change in pressure over a 120 s hold (Fig. 1D). As illustrated, hysteresis was calculated as the difference in area between the inflation and deflation portion of the PV curve with respect to the y-axis at volumes of 0.5, 0.7, 0.9 ml. Energy Loss was calculated as the normalized area γ and the sum of area γ & β to account for the increase of hysteresis with increased inflation volume (Fig. 1E)^38^.

For each of the preceding calculations, the results from all five mice were averaged, analyzed for statistical significance across volumes and cycling rates, and reported as mean ± standard deviation (Table 1). Damaged or leaky specimens were omitted from analysis affecting sample size for percent relaxation measurements alone (n ≥ 3). Statistical analysis was conducted using nonparametric Friedman’s Test in GraphPad Prism 9 (Version 9.1.0, GraphPad Software, San Diego, CA, USA). Post-hoc analyses with Dunn’s multiple comparison test were used to test pairwise differences. Significance was defined as p < 0.05 with *p < 0.05, **p < 0.01, ***p < 0.001, ****p < 0.0001.

**Table 1:** Values of static compliance (C), peak pressure, starting compliance (C_start_), inflation compliance (C_inf_), top compliance (C_top_), deflation compliance (C_def_), percent relaxation, hysteresis, and energy loss as mean ± standard deviation reported for variable applied volume loading at corresponding breathing rates.

Breaths per minute	Applied volume (ml)	C (µl/cm H_2_O)	Peak pressure (cmH_2_O)	C_start_ (µl/cm H_2_O)	C_inf_ (µl/cm H_2_O)	C_top_ (µl/cm H_2_O)	C_def_ (µl/cm H_2_O)	Percent relaxation (%)	Hysteresis (ml·cmH_2_O)	Energy loss (%)
5 BPM	0.3	6.9 $\pm$ 1.1	17.76 $\pm$ 0.29	4.8 $\pm$ 1.5	–	3.6 $\pm$ 2.1	12.4 $\pm$ 2.8	21.7 $\pm$ 4.6	–	–
	0.5	8.5 $\pm$ 0.9	26.37 $\pm$ 1.33	5.5 $\pm$ 0.6	–	4.2 $\pm$ 1.8	11.3 $\pm$ 0.7	27.5 $\pm$ 1.8	1.35 $\pm$ 0.23	41.2 $\pm$ 3.0
	0.7	12.3 $\pm$ 1.4	30.39 $\pm$ 2.28	5.7 $\pm$ 0.9	30 $\pm$ 7.8	6.0 $\pm$ 1.6	16.6 $\pm$ 1.6	37.3 $\pm$ 7.4	3.61 $\pm$0.09	48.0 $\pm$ 2.0
	0.9	16.1 $\pm$ 2.3	33.79 $\pm$ 3.02	7.0 $\pm$ 1.0	33.7 $\pm$ 11.0	7.4 $\pm$ 1.5	22.2 $\pm$ 2.2	32.3 $\pm$ 5.5	6.16 $\pm$ 0.39	49.7 $\pm$ 2.5
10 BPM	0.3	6.8 $\pm$ 0.6	18.13 $\pm$ 0.52	6.1 $\pm$ 0.8	–	3.0 $\pm$ 1.5	8.4 $\pm$ 1.5	28.1 $\pm$ 10.0	–	–
	0.5	7.7 $\pm$ 0.7	26.98 $\pm$ 1.30	6.1 $\pm$ 0.8	–	4.6 $\pm$ 1.9	10.8 $\pm$ 0.4	32.7 $\pm$ 5.9	1.17 $\pm$ 0.16	39.0 $\pm$ 3.6
	0.7	11.8 $\pm$ 1.3	31.19 $\pm$ 2.31	5.3 $\pm$ 1.0	40.7 $\pm$ 25.9	5.8 $\pm$ 1.9	16.4 $\pm$ 1.6	37.0 $\pm$ 4.2	3.91 $\pm$ 0.21	51.2 $\pm$ 1.5
	0.9	14.7 $\pm$ 1.6	35.38 $\pm$ 2.27	7.2 $\pm$ 0.9	34.9 $\pm$ 9.1	7.5 $\pm$ 1.5	21.6 $\pm$ 1.8	36.6 $\pm$ 5.4	6.51 $\pm$ 0.42	52.8 $\pm$ 2.3
20 BPM	0.3	5.4 $\pm$ 0.5	18.80 $\pm$ 0.59	6.1 $\pm$ 1.1	–	2.9 $\pm$ 0.8	5.3 $\pm$ 0.6	24.7 $\pm$ 4.0	–	–
	0.5	6.6 $\pm$ 0.8	28.64 $\pm$ 1.48	5.8 $\pm$ 0.7	–	3.5 $\pm$ 1.0	9.0 $\pm$ 0.9	34.4 $\pm$ 2.0	1.11 $\pm$ 0.12	41.4 $\pm$ 2.3
	0.7	10.1 $\pm$ 0.9	33.79 $\pm$ 1.60	5.9 $\pm$ 0.8	25.6 $\pm$ 1.1	5.3 $\pm$ 1.1	14.7 $\pm$ 1.1	39.4 $\pm$ 4.4	4.02 $\pm$ 0.30	52.8 $\pm$ 1.4
	0.9	13.2 $\pm$ 1.6	37.72 $\pm$ 2.60	7.3 $\pm$ 1.9	35.6 $\pm$ 10.2	6.3 $\pm$ 0.7	21.2 $\pm$ 3.0	39.2 $\pm$ 7.1	7.6 $\pm$ 0.66	58.1 $\pm$ 2.4

## Results

### Hysteresis and energy loss

Hysteresis was found to significantly increase as inflation volume increased across all four tested volumes. After normalization, energy loss was found to significantly vary with inflation volume across a single volume transition between 0.5 to 0.9 ml at all cycling rates (5 BPM, p = 0.0047; 10 BPM, p = 0.0047; 20 BPM, p = 0.0047).

**Figure 2:**
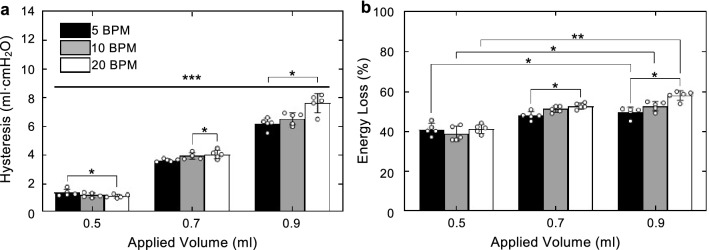
Values plotted as mean ± standard deviation with open circles indicating individual specimen values. (**a**) Hysteresis increased significantly with inflation volume. (**b**) Despite normalization, energy loss increased significantly with an increase in inflation volume from 0.3 to 0.9 ml. For both hysteresis and energy loss, an increase in cycling rate caused a significant increase in hysteresis and energy loss respectively: hysteresis increased at 0.5 ml between 5 and 20 BPM, at 0.7 ml between 10 and 20 BPM, and at 0.9 ml between 5 and 20 BPM; energy loss increased at 0.7 ml between 5 and 20 BPM and at 0.9 ml between 5 and 20 BPM.

It was found that hysteresis was significantly affected by a change in cycling rate at the transition between 5 to 10 BPM at 0.7 ml (p = 0.0342) and the transition between 5 to 20 BPM at 0.5 ml (p = 0.0342) and the highest inflation volume of 0.9 ml (p = 0.0342). Energy loss behaved similarly, increasing significantly between 5 and 20 BPM when inflation volume was 0.7 (p = 0.0342) and 0.9 ml (p = 0.0133), as shown in Fig. 2.

### Compliance

**Figure 3:**
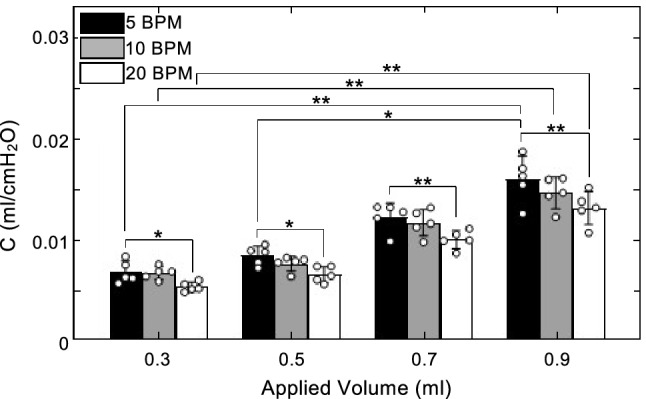
Static compliance plotted as mean ± standard deviation with open circles indicating individual specimen values. C significantly increased with inflation volume and decreased with cycling rate. Increased volume from 0.3 to 0.9 ml resulted in significantly increased compliance at all three rates. Volume changes from 0.5 to 0.9 ml increased C significantly at 5 BPM. A significant decrease in C occurred between 5 and 20 BPM for all four volumes.

Static compliance was found to increase with inflation volume (Fig. 3). This trend was found to be significant at the following junctions: first, as inflation volume increased from 0.3 to 0.9 ml at all the rates (5 BPM, p = 0.0036; 10 BPM, p = 0.0014; 20 BPM, p = 0.0014) and as inflation volume was increased from 0.5 to 0.9 ml at 5 BPM (p = 0.0423).

Static compliance was also found to decrease with increasing cycling rate. The decrease was found to be significant when the cycling rate increased from 5 to 20 BPM at all four volumes (0.3 ml, p = 0.0342; 0.5 ml, p = 0.0133; 0.7 ml, p = 0.0047; 0.9 ml, p = 0.0047).

**Figure 4:**
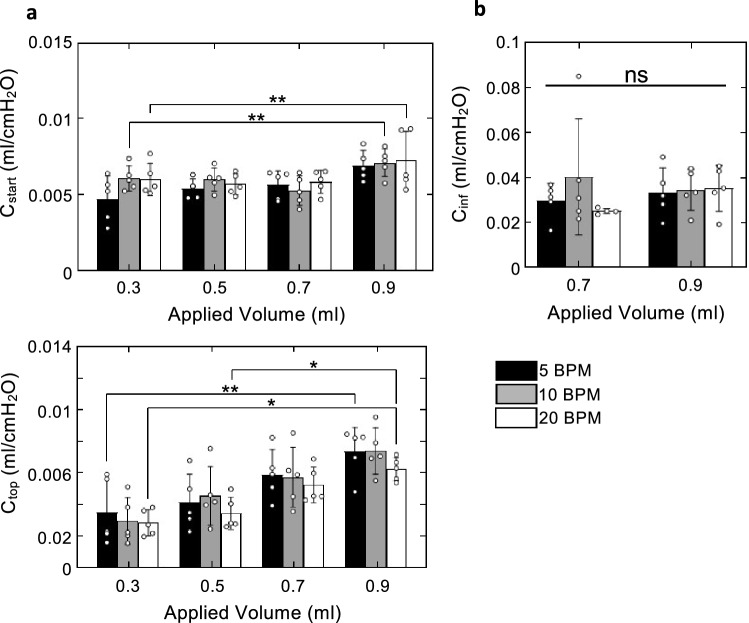
C_start_, C_inf_, C_top_ values are plotted as mean ± standard deviation with open circles indicating individual specimen responses. (**a**) C_start_ significantly varied with volume from 0.3 to 0.9 ml and from 0.5 to 0.9 ml at 5 BPM, and from 0.7 to 0.9 ml at 10 BPM. C_start_ did not significantly vary with cycling rate. (**b**) C_inf_ did not significantly vary with neither inflation volume nor cycling rate. (**c**) C_top_ increased with applied volume significant from 0.3 to 0.9 ml at all three rates and from 0.5 to 0.9 ml at 5 BPM. C_top_ decreased with cycling rate, but the trend was not statistically significant.

Starting compliance, inflation compliance, and top compliance were not significantly affected by cycling rate. Starting compliance and top compliance were significantly affected by inflation volume. C_start_ increased from 0.3 to 0.9 ml (p = 0.0423) and from 0.5 to 0.9 ml at 5 BPM (p = 0.0423), and from 0.7 to 0.9 ml at 10 BPM (p = 0.0197). C_top_ increased from 0.3 to 0.9 ml at all three rates (5 BPM, p = 0.0087; 10 BPM, p = 0.0087; 20 BPM, p = 0.0087) as well as from 0.5 to 0.9 ml at 5 BPM (p = 0.0197) as shown in Fig. 4.

**Figure 5:**
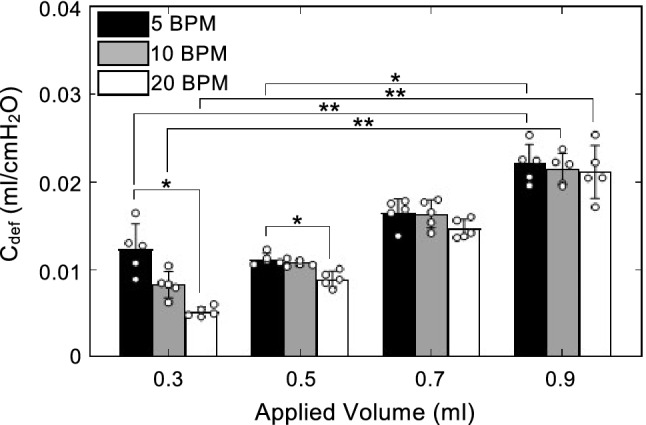
Deflation compliance plotted as mean ± standard deviation with open circles indicating individual specimen values. C_def_ significantly increased with max inflation volume and decreased with cycling rate. Increased volume from 0.3 to 0.9 ml increased C_def_ at all three rates. Increased volume from 0.5 to 0.9 ml resulted in a significant increase in C_def_ at 5 BPM. An increase in cycling rate from 5 to 20 BPM was found to be significant at lower inflation volumes.

Deflation compliance, as shown in Fig. 5, tended to decrease as cycling rate increased. As the volume increased, this dependence lessened and the only significant dependence on BPM was observed to occur at the three smallest inflation volumes between 5 and 20 BPM (0.3 ml, p = 0.0047; 0.5 ml, p = 0.0047; 0.7 ml, p = 0.0342). Deflation compliance was found to significantly increase between 0.3 and 0.9 ml at all three rates (5 BPM, p = 0.0423; 10 BPM, p = 0.0014; 20 BPM, p = 0.0014), and between 0.5 and 0.9 ml at 5 BPM (p = 0.0036).

### Percent relaxation

**Figure 6:**
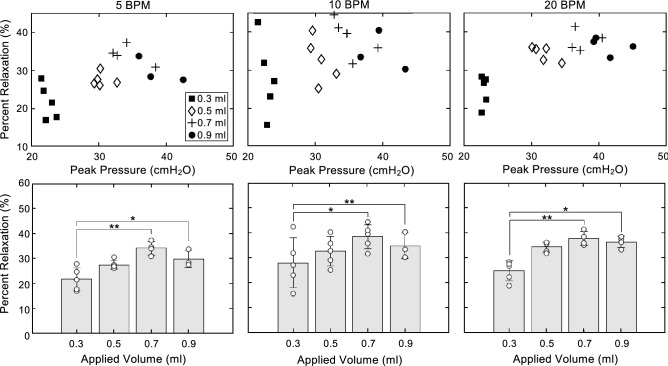
Markers indicate individual specimen’s percent relaxation as a function of peak pressure before the hold (top row) or applied inflation volume (bottom row). Percent relaxation at low peak pressures varied more than percent relaxation at higher pressures. Breathing frequencies of 5 and 20 BPM cluster around 30% and 35% respectively after reaching pressures of 30 cmH_2_O. Percent relaxation varied significantly with increasing inflation volume from 0.3 to 0.7 ml and 0.3 to 0.9 ml for all three rates. Additionally, percent relaxation varied significantly with increasing cycling rate from 5 to 20 BPM at 0.5, 0.7, and 0.9 ml.

Percent relaxation increased over the range of 0.3 to 0.7 ml and decreased from 0.7 to 0.9 ml. This change was found to be statistically significant at two transitions from 0.3 to 0.7 ml (5 BPM, p = 0.0036; 10 BPM, p = 0.0197; 20 BPM, p = 0.0087) and from 0.3 to 0.9 ml (5 BPM, p = 0.0423; 10 BPM, p = 0.0087; 20 BPM, p = 0.0423) at all three rates. Increasing cycling rate significantly increased percent relaxation from 5 to 20 BPM at 0.5, 0.7, 0.9 ml (0.5 ml, p = 0.0047; 0.7 ml, p = 0.0047; 0.9 ml, 0.0400). For 5, 10, and 20 BPM, the percent relaxation at lower peak pressures showed a greater specimen-to-specimen variability than at higher pressures. Furthermore, at 5 and 20 BPM when the pressures were higher than ~ 30 cmH_2_O, the measurements of percent relaxation clustered around 30–35% (Fig. 6).

### Peak pressure

**Figure 7:**
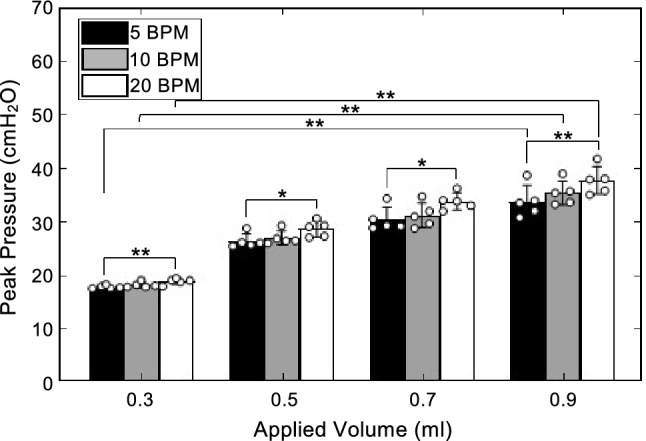
Peak pressure was plotted as mean ± standard deviation with open circles indicating individual specimen responses. Peak pressure increased significantly with inflation volume from 0.3 to 0.9 ml at all three rates. Increased cycling rate had a significant effect on peak pressures at all four volumes when increased from 5 to 20 BPM.

Increasing the cycling rate significantly affected the peak pressure at all four volumes from 5 to 20 BPM (0.3 ml, p = 0.0047; 0.5 ml, p = 0.0133; 0.7 ml, p = 0.0133; 0.9 ml, p = 0.0047). Increasing inflation volume resulted in an increase in peak pressure from 0.3 to 0.9 ml at all three rates (5 BPM, p = 0.0014; 10 BPM, p = 0. 0014; 20 BPM, p = 0. 0014) (Fig. 7).

## Discussion:

This study systematically analyzes mouse lung mechanics as both the inflation volume and cycling rate are varied. Viscoelastic measures were conclusively rate dependent; and while hysteresis significantly increased with inflation volume, stress relaxation tended to increase but subsequently decreased at 0.9 ml. Of the five compliance measures analyzed, static compliance and deflation compliance increased with inflation volume and decreased with increased cycling rate; starting and top compliance also increased with volume while inflation compliance was not volume dependent. Additionally, starting, inflation, and top compliance did not vary with cycling rate.

The maximum inflation pressure and volume influence the final shape of the PV curve. At lower volumes, the PV curve forms a classical crescent shape within the physiological elastic realm while at larger volumes it is a double sigmoidal curve^29^. The PV curves in our study adhere to this trend (Fig. 1B). In vivo studies have found the transition of the crescent to the double sigmoidal PV curve to be past 20–25 cmH_2_O^29^; in our study the transition occurs past slightly higher pressures between 26–30 cmH_2_O, between 0.5 and 0.7 ml applied volumes. This slight inter-study variability may be due to difference in age and or strain of mice studied. One proposed cause of this shape change is the recruitment of a second set of alveoli. Namati et al. showed the appearance of new alveoli via the pores of Kohn at pressures above 25 cmH_2_O, a form of collateral ventilation, believed to have previously been closed off by surfactant^39^. Zosky et al. supported this explanation via an in vivo study on elastance over a range of inflation pressures^29^. In our ex vivo study, we observe trends supporting this theory as well: between the lowest and highest inflation volumes and after the transition volume occurred, C increased, C_inf_ appeared, and energy loss increased. The increase in static compliance is unexpected because it indicates a softer lung that is easier to inflate at higher volumes. Lung tissue is known to experience strain-stiffening which would cause a decrease in static compliance, a stiffer lung, at higher inflation volumes^40,41^; this effect has been demonstrated on the surface of the murine lung during inflation at 0.7 and 0.9 ml using digital image correlation, which allows analysis of simultaneous global and local behavior^42,43^. However, increasing the internal space available within the lung, as caused by the opening of a secondary (daughter) set of alveoli, would create more regions for air migration, alleviating the excess strain on alveoli and thus increasing the static compliance^36,44^. The sudden availability of a newly engaged set of alveoli would alter the direction of the PV curve by decreasing the effort needed to add air causing the knee in the inflation limb and the appearance of C_inf_. The increase in energy loss could also be due to the opening of this secondary set of alveoli, as additional energy would be required to open this subsequent set of alveoli.

PV curves for the mouse are typically collected over the full range of the lung, where the lung is first degassed so that it starts at zero volume and then inflated above the aforementioned transitional pressure to total lung capacity (~ 30–35 cmH_2_O)^24,25^. This degassing step causes potentially damaging alveolar collapse, requiring greater reopening pressures and alters the lung mechanics of damaged lungs^45,46^. To avoid alveoli and airway collapse, the measurements are standardized by a preload pressure in this study^26^. Comparing our collected curves to degassed PV curves, such as those collected by Robichaud and Limjunyawong, reveals expected differences in the start of inflation limb^24,25^. The previously reported slope of this limb in healthy degassed specimens ranged from 2.2–4.2 µl/cmH_2_O, whereas the starting compliance in our study is greater, ranging from 4.8–7.3 µl/cmH_2_O^5^. The discrepancy is most likely the consequence of not degassing, for which our findings reveal more compliant lung behavior that is easier to inflate.

We find that inflation volume affects starting compliance while cycling rate has no effect. In contrast to our findings, other studies show that starting compliance did not vary with inflation pressure nor inflation volume^36,47^. Previously, the starting compliance of non-degassed lungs has been thought to be a representation of the number of “lung units'' available or the amount of aerated tissue before inflation^36,48^. Namati et al. found that when inflating a non-degassed mouse lung from 5 to 20 cmH_2_O, which is within the range of our starting compliance curve, open alveoli expanded but did not increase in number^39^. Interestingly, our findings suggest that the starting compliance is affected by inflation volume despite this variable not influencing the number of lung units available at the start of inflation.

Static compliance (C) has been found to decrease with increased breathing frequency in dogs and cats and with increased inflation rate in mice^26,49–52^. The same trend has also been observed for dynamic compliance in diseased human lungs and is thought to be a means of diagnosis^50–54^. In agreement with previous literature, our study finds that static compliance tends to decrease with increased frequency (Fig. 7). Grotberg and Davis also observed frequency dependence in healthy ex vivo dog lobes, hypothesizing these trends may be attributed to the isolation of the specimens and lack of chest wall^31^. Our study observes a small frequency dependence in the whole organ of a healthy mouse lung which may diminish if the lungs are intact and within the chest cavity. As such, the frequency dependence of the ex vivo lungs may, in part, be due to the viscoelastic properties of lung tissue^55,56^.

The deflation compliance (C_def_) is often used in murine studies to measure the compliance of the lung as opposed to C^24,25,57,58^. C_def_ in our study exhibited trends most comparable to those of C. That is, both C_def_ and C experience a decrease with increasing cycling rate and an increase with increasing inflation volume. However, the volume dependency of the effect of cycling rate was opposite for the two slopes: as volume increased, C_def_ became nondependent on frequency, while C became more dependent on frequency as the volume increased. Takeuchi found that C_def_ and C_inf_ in sheep experienced the same trends and were equal at all values^36^. That study linked the values of C_inf_ and C_def_ to the rate of recruitment and decruitment, respectively. In our mouse study, C_inf_ was not present at the lower volumes but its appearance correlated with a statistically significant increase in C_def_ across cycling rates. Given the potential for C_inf_ to be representative of the opening of a secondary set of alveoli^39^ as discussed earlier, an increase of decruitment after its appearance at higher volumes is unavoidable. The increase of C_def_ after the appearance of C_inf_ could indicate the increased decruitment of lung units. However, C_def_ continues to increase with volume while C_inf_ is unaffected, unlike previous findings.

Hysteresis is caused by the surface tension of surfactant and the viscoelastic nature of lung tissues and is an important focus in disease modeling^17,27,55,59^. It has previously been observed that hysteresis increases slightly with frequency in dogs^49^. We find, in agreement with previous studies, that hysteresis significantly increases with cycling rate (significant for 0.5 and 0.9 ml between 5 and 20 BPM and 0.7 ml between 10 and 20 BPM). Further, it was observe in this study that the effect of cycling rate on hysteresis is slightly volume dependent: at a lower volume, 0.5 ml, hysteresis tends to slightly decrease with increasing frequency, while at higher volumes the effect of frequency reverss and causes an increase in hysteresis. This agrees with Grotberg’s findings at higher volumes, but Grotberg did not note variations in hysteresis at lower volumes as we do. In contrast, Hildebrandt found hysteresis to not have any volume dependency when examining frequency dependence in cat lungs; Grotberg hypothesized the disagreement in findings can be attributed to Hildebrandt examining the whole lung, as opposed to testing the lobes individually, which allowed for a variety of air distributions and eliminated this trend. Interestingly, in this study we test the whole organ ex vivo as Hildebrandt did but find the volume dependency seen by Grotberg. We also find frequency trends between hysteresis and energy loss (normalized hysteresis based on loading) were comparable. Although these trends are minimal, insights are limited by the range of cycling rates since physiological breathing rates in mice range from 250–350 BPM^18^ and the speed is limited by the apparatus, despite being faster than past works^25^. As such, variabilities between these studies and frequency dependencies, along with isolated airway and parenchymal strips demonstrating loading dependent hysteresis variabilities, merits further exploration at expanded breathing rates ranges^17,55^.

Viscoelastic measures of the whole organ, such as entropy, are demonstrated to be beneficial markers of diseases^27^. However, the viscoelastic parameter of percent relaxation of the whole organ is understudied. This viscoelastic relaxation of the whole lung results, in part, from the stress relaxation of the airways and parenchyma^17,32,60^^.^ The shape of our viscoelastic relaxation curve after holding a fixed inflation volume consists of an initial drop in pressure followed by a slower asymptotic decay, as previously demonstrated in mice^26,61^. Trends of this viscoelastic hold have been studied in response to changes in body temperature, acute blood volume expansion, and interleukin IL-6^62–64^. The effect of inflation volume has been investigated in rabbits and briefly in mice, both studies found an increase in stress relaxation with volume; for rabbits this trend continued up to the point of rupture^26,65^. We observe this significant tendency up to an inflation volume of 0.7 ml, after which, at an increased volume of 0.9 ml, we note a decrease in percent relaxation; this decrease has not been previously reported and does not align with the findings from rabbits. Observations may be attributable to the difference in species (e.g., collateral ventilation, smooth muscle orientation^66–68^), the analysis of the length of the viscoelastic hold (~ 1 s versus 120 s), the inclusion of the initial drop in pressure as opposed to only the asymptotic decay^65^, or potentially unidentified leaks in our study at this higher volume.

## Limitations

The lung specimens used in this study were not degassed. While this restricts comparative insights, it allows the physiological assessment of lung behavior, instead of forcing the reinflation of collapsed airways and alveoli that is seen in degassing. Additionally, while the tissue hydration was maintained, the testing protocols were several hours in duration which may cause deterioration, albeit this is assumed to be negligible. Leaks also unavoidably developed in specimens and reduced the number of samples available for analysis. Lastly, while the shape of the in vivo and ex vivo PV curve is similar, our ex vivo experiments do not incorporate the role of the chest cavity which may influence the absolute measurement values collected here.
